# Clinical Outcomes, Healthcare Utilization, and Cost Following Implementation of a High‐Sensitivity Cardiac Troponin Assay

**DOI:** 10.1002/clc.70133

**Published:** 2025-05-06

**Authors:** Indu Poornima, Teigan Dwyer, Tyson Barrett, Tyler Moore, Corey Clarke, Brent A. Williams

**Affiliations:** ^1^ Allegheny Health Network Pittsburgh Pennsylvania USA; ^2^ Highmark Health Pittsburgh Pennsylvania USA

**Keywords:** chest pain evaluation, emergency department, high‐sensitivity troponin

## Abstract

**Introduction:**

High‐sensitivity troponin (Hs‐trop) protocols have been developed for the cardiac evaluation of chest pain patients presenting to emergency departments (ED), but uptake has been suboptimal.

**Methods:**

This retrospective study sought to evaluate the effects of an Hs‐trop protocol (Roche Gen‐5 troponin assay) implementation on patient outcomes, healthcare utilization, and costs. Patients presenting to EDs with chest pain following implementation of an Hs‐trop protocol (POST) were compared to control patients presenting in the year prior (PRE). Study endpoints included troponin elevations, cardiac diagnostic testing, and ED disposition. Among patients discharged directly from the ED, 30‐ and 90‐day death, new myocardial infarction (MI), ED returns, and hospital admissions were compared. In a subset with insurance data, post‐discharge healthcare costs and utilization were compared.

**Results:**

Among 15 015 patients meeting study criteria, there were no differences in MI diagnoses POST versus PRE, but myocardial injury without MI was more frequent POST (aOR = 9.03; 95% CI: 7.44, 10.96). Noninvasive cardiac testing at the index ED encounter was less frequent POST (aOR = 0.72; 0.67, 0.78), with no difference for invasive angiography. Among patients directly discharged from the ED, no differences were observed for death, but POST patients had fewer ED returns (aOR = 0.70; 0.59, 0.83) and hospital admissions (aOR = 0.62; 0.45, 0.85) within 30 days. Overall healthcare utilization was 8.4% lower in the POST group (*p* < 0.001) but costs were not different.

**Conclusions:**

Following implementation of an Hs‐trop protocol, decreases were observed in noninvasive cardiac testing, and ED returns and hospital admissions within 90 days of discharge, without compromising outcomes. Overall healthcare utilization declined.

## Graphical abstract

Following implementation of a high‐sensitivity troponin (Hs‐trop) protocol, decreases were observed in noninvasive cardiac testing, and emergency department (ED) returns and hospital admissions within 90 days of discharge, without compromising outcomes. Overall healthcare utilization declined.

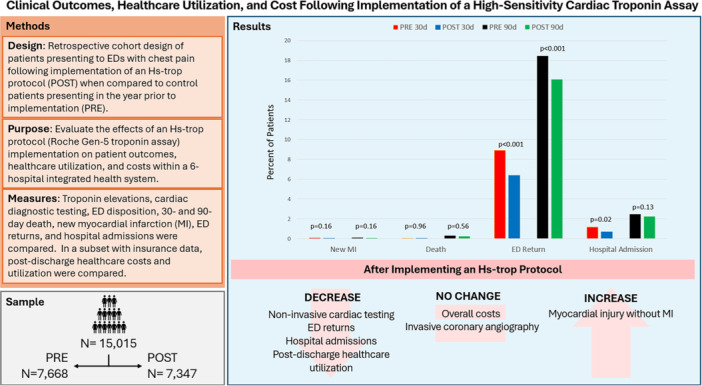

## 1 Introduction

High‐sensitivity troponin (Hs‐trop) was introduced in the United States (US) in 2017 and its adoption has been steadily increasing across US hospitals [1, 2]. The 2021 ACC/AHA guidelines for chest pain evaluation endorsed transition to Hs‐trop to appropriately triage patients with chest pain [3]. Still, as of 2021 only 34% of the 550 hospitals in the National Cardiovascular Data Registry – Chest Pain‐MI registry had made the transition to Hs‐trop [4]. Data from this registry found that transition to Hs‐trop did not change rates of stress testing or coronary computed tomography angiography (CCTA) in low risk patients, but did lower rates of invasive angiography [4]. International studies and reports from integrated US health systems have found higher rates of emergency department (ED) discharges and reduced length of stay (LOS) in the ED and hospital after transition to Hs‐trop [5, 6, 7, 8]. However, impact on cost of care has been variable with international studies reporting significant cost reduction while comparable studies in the US have not demonstrated similar results [5, 6, 7, 8].

The majority of published implementation studies in the US have focused on in‐hospital outcomes with few reporting on longer‐term follow up of patients directly discharged from EDs after implementation of an Hs‐trop T assay [9]. Favorable post‐discharge outcomes should increase the confidence of ED providers and stimulate change in the US healthcare environment. The potential impact on cost of care has implications for health systems considering this transition as a more sensitive test could lead to cascade testing and higher cost of care [10, 11] This data is relevant in the current healthcare landscape with transition to payment models where healthcare systems participate in partial or full risk contracts to address costs of care and is important to patients who bear a higher portion of total care costs [12]. Accordingly, the present study, conducted across a 6‐hospital integrated health network that includes an academic quaternary and five community hospitals, evaluated the effect of implementing an Hs‐trop pathway among patients presenting to the ED with chest pain on the following metrics: (1) rates and patterns of cardiac testing at the index ED presentation; (2) rates of hospitalization and direct ED discharge; (3) LOS in the ED and hospital; (4) 30‐ and 90‐day mortality and return ED visits among those directly discharged from the ED; and (5) in a subset with insurance data, post‐discharge cost of care and healthcare utilization.

## 2 Methods

This retrospective study included consecutive patients that presented to EDs with a primary complaint of chest pain or other symptoms suspicious for acute coronary syndromes (ACS) at any of six hospitals associated with the Allegheny Health Network (AHN), an integrated health network located in western Pennsylvania. Patients ≥ 18 years of age presenting during the study period were included if they had at least one troponin measured in the ED and a primary billing diagnosis indicative of chest pain, defined by any of the following International Classification of Diseases 9th or 10th edition (ICD‐9/10) codes: 786.5, 786.59, R07.82, R07.89, R07.9, I20, I21, or I22. Thereafter, patients were assigned to pre‐ or post‐implementation groups (PRE vs. POST) according to whether ED presentation was in the year before or following implementation of an Hs‐trop protocol. Protocol initiation dates varied across AHN hospitals. At Allegheny General Hospital – the academic quaternary hospital of AHN – protocol implementation began April 20, 2021, while at the community hospitals protocol implementation began either September 16 or October 14, 2021. For patients with multiple ED encounters for chest pain during the study period, only the first encounter was considered. This study was approved by the AHN Institutional Review Board which granted a waiver of patient consent.

### 2.1 Chest Pain Evaluation Pathways

**Figure 1:**
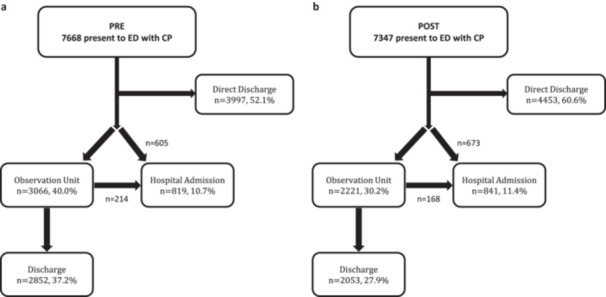
(a and b) Emergency department disposition by pre‐ versus post‐implementation (PRE vs. POST) of a high‐sensitivity troponin protocol.

Chest pain assessment considered quality of chest pain, presence of ischemic EKG changes, and troponin values as specified by the Universal Definition of Myocardial Infarction [13]. Before April 2021, evaluation of chest pain in AHN EDs included assessment of the HEART score and measurement of one or more Gen‐4 troponin levels (Roche Gen‐4 assay) (Supporting Information S1: Figure 1). Both the limit of detection and 99th percentile cutoff for the Gen‐4 assay was 0.01 ng/ml. In the pre‐implementation phase, patients with HEART scores of 0–3 were deemed low risk and discharged from the ED if two Gen‐4 troponin levels, drawn 6 h apart, were < 99th percentile values. Patients with HEART scores of 4–7 (intermediate) and > 7 (high) were admitted to either Observation or Inpatient units for further testing based on EKG findings and symptoms. All patients with low HEART scores discharged from the ED had cardiology follow up within 72 h.

### 2.2 Hs‐Troponin Transition Implementation Process

**Figure 2:**
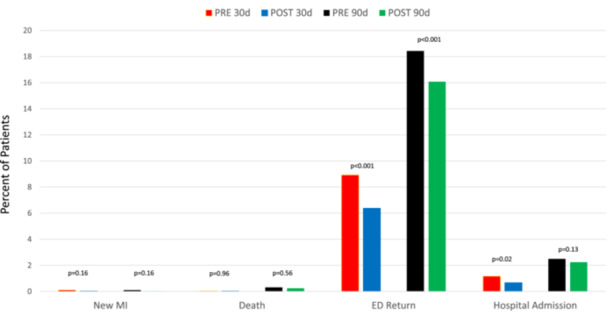
Rates of 30‐ and 90‐day post‐discharge myocardial infarction (MI), death, emergency department (ED) return, and hospital admission among patients directly discharged from the ED, stratified by pre‐ versus post‐implementation (PRE vs. POST) of a high‐sensitivity troponin protocol.

Between April and October 2021, each AHN hospital transitioned to the Roche Gen‐5 Hs‐trop assay for cardiac evaluation of chest pain/suspected ACS patients. Transition planning began a year before transition with internal validation of the Roche Gen‐5 troponin assay by AHN Lab Medicine. A multidisciplinary team consisting of Cardiology, Emergency Medicine and Hospitalist physicians, along with Nursing and Laboratory Medicine was then assembled. The Hs‐trop chest pain decision pathway (CDP) at AHN was created after multiple deliberative engagement sessions with all stakeholders (Supporting Information S1: Figure 2). Prior studies and published institutional protocols were considered when creating the Hs‐trop CDP that also included the HEART score without troponin (HEAR) [2, 9, 14]. The HEAR score categories were similar to the HEART score. The salient features of the protocol were: (1) a shorter duration between the 1st and 2nd Hs‐trop measurements; (2) measurement of Hs‐trop before consideration of the HEAR score; (3) use of delta Hs‐trop to assess the significance of an elevated Hs‐trop; (4) patients discharged from the ED with negative Hs‐trop and low risk HEAR scores followed up primarily with a primary care provider with optional cardiology follow‐up; and (5) patients with intermediate HEAR scores (4‐7) with negative Hs‐trop were either admitted to Observation or had stress testing or CCTA in the ED and could be discharged directly from the ED.

Extensive system‐wide education occurred before implementations of the protocol in the form of Special Grand Rounds, mandated for all stakeholder departments including trainees. In addition, case‐based educational sessions were made available to nursing. These sessions were recorded and made available on the intranet. The assay was initially made available only to Emergency Medicine and Cardiology attendings at the hub academic center alongside the Gen‐4 troponin assay. The short period of overlap between both assays from September to December 2020 facilitated understanding of the results of the Hs‐trop assay in the various clinical contexts, in comparison to the Gen‐4 troponin assay. Results and downstream clinical care were assessed for 2 months and feedback solicited from all stakeholders. Minor changes to the electronic orders were implemented based on the feedback. Among the six AHN hospitals implementing the Hs‐trop protocol, the total bed capacity of all hospitals combined is 1950 and the number of ED visits in 2021 was approximately 240 000.

### 2.3 Study Outcomes

Multiple cardiac and diagnostic testing endpoints occurring in association with the presenting ED encounter were identified and compared across PRE and POST groups. For the Roche Hs‐trop assay, *troponin elevation* was defined as exceeding the sex‐specific 99th percentile of the assay (14 ng/L for women; 22 ng/L for men); the limit of detection (LOD) was 6 ng/L. These values were the FDA approved cutoffs for this assay. A value > 52 ng/L was classified as acute myocardial infarction and a difference > 6 ng/L between consecutive assays was considered a significant change. For the Gen‐4 troponin‐T assay, troponin elevation was defined as ≥ 0.01 ng/ml for both sexes based on the manufacturer's recommendation. Diagnoses of myocardial infarction (MI) associated with the index ED encounter were identified via ICD‐10 diagnosis codes in the EHR. MI diagnoses were differentiated into Type 1 versus Type 2 based on EHR documentation according to definitions established in the Fourth Universal Definition of Myocardial Infarction and included a combination of symptoms, electrocardiography, and troponin assessment [13]. Type 1 MI was further differentiated into ST‐elevation MI (STEMI) or non‐STEMI (NSTEMI) based on EKG changes. Patients with elevated troponins but not otherwise meeting MI diagnostic criteria were classified as *myocardial injury* without infarction and included those with acute (e.g., myocarditis) and chronic (e.g., kidney disease) causes.

### 2.4 Disposition, Resource Utilization, and Quality Metrics

Patient baseline characteristics including demographics and medical history were determined through historical encounters documented in the AHN EHR using all available information before and including the initial ED encounter. Performance of echocardiography, stress testing, CCTA, and invasive coronary angiography during the index ED encounter were determined within PRE and POST groups from EHR documentation of Current Procedural Terminology 4th edition codes. Rates of cardiac testing were also compared between the quaternary care hospital (AGH) and community hospitals combined (non‐AGH) to detect possible variations. ED disposition was classified as direct discharge, admission to hospital, or observation unit. Length of stay in the ED and hospital were measured in hours. Among patients discharged from the ED without MI or troponin elevation, termed *early discharge*, multiple clinical endpoints were tracked through the AHN EHR within 30 and 90 days following ED discharge, including all‐cause mortality, new MI diagnoses, ED return for any reason, and hospital admission for any reason.

Among the subset of study patients with concomitant membership in the AHN‐affiliated Highmark Health Plan (HHP) at the time of index ED presentation, multiple healthcare utilization and cost metrics were tracked following discharge. Inclusion in this subset required continuous HHP enrollment in the 3 months before the initial ED presentation and 3 months following discharge. Among this subset, costs were calculated for both the 3 months preceding ED presentation as well as the 3 months following ED discharge. Furthermore, healthcare utilization (i.e., number of encounters) per member was calculated for both baseline and post‐discharge periods. Both costs and healthcare utilization are reported overall and separately by claim type as outpatient, inpatient, professional, or drug prescription. All cost and utilization metrics are reported as per member per month.

### 2.5 Statistical Analysis

To assess baseline comparability of the PRE and POST groups, descriptive statistics were compared with binary variables reported as frequencies and percentages, and continuous variables as medians with interquartile ranges. Binary characteristics were compared across implementation groups with chi‐square tests, and continuous baseline characteristics with Wilcoxon rank sum tests. Differences in binary endpoints such as percentage of patients with troponin elevation, performance of cardiac diagnostic testing, cardiac diagnoses, and ED disposition across PRE/POST groups were evaluated using logistic regression with and without adjustment for important demographic and medical history variables. Adjusted odds ratios (aOR) and 95% confidence intervals are reported comparing POST to PRE groups. Thirty‐ and 90‐day post‐discharge outcomes were also defined as binary and differences across study groups assessed via logistic regression with and without adjustment.

For the HHP subset, in the 3‐month period following discharge, healthcare utilization and cost of care were compared between study groups. Large outliers were trimmed at three standard deviations above the mean to lessen their influence on statistical findings. Unadjusted utilization and costs are reported both as medians with interquartile ranges and means with standard deviations. Inferential comparisons were done via two analyses: (1) Wilcoxon rank sum tests, and (2) ordinary least squares regression, with natural log transformations of the dependent variables given the right skew of cost and utilization measures. For regression analyses, adjusted models controlled for baseline utilization/costs and important demographic and medical history variables. Regression coefficients (β) from models with log transformed utilization/costs are interpreted as the relative percentage difference (increase/decrease) in utilization/costs in the POST versus PRE groups. Analyses were done using SAS version 8.2 and R version 3.6.0.

## 3 Results

Across all six AHN hospitals, 15 015 patients presented to the ED with chest pain or other symptoms suspicious for ACS and met other study criteria (PRE *n* = 7668; POST *n* = 7347). Study patients had a median age of 55 years and were more commonly of White race (77%) and female (54%). No significant differences in patient demographics were observed across PRE and POST groups (Table 1). Patients in the POST group had a slightly greater documented history of obesity (42.0% vs. 36.2%), tobacco use (20.4% vs. 16.0%), and heart failure (10.4% vs. 9.3%), but less documented history of dyslipidemia (40.4% vs. 45.9%) and cerebrovascular disease (6.9% vs. 8.4%). Prior history of coronary artery disease (20.5%) was similar between groups.

**Table 1:** Baseline characteristics of patients presenting to the emergency department with a primary complaint of chest pain stratified by pre‐ versus post‐implementation of a high‐sensitivity troponin protocol.

	All (*n* = 15 015)	PRE (*n* = 7668)	POST (*n* = 7347)	*p*‐value
Age	54.9 (40.1, 67.5)	55.0 (40.1, 67.3)	54.7 (40.0, 67.6)	0.82
Female	8129, 54.1%	4122, 53.8%	4007, 54.5%	0.34
White race	11596, 77.2%	5946, 77.5%	5650, 76.9%	0.40
Obesity	5860, 39.0%	2776, 36.2%	3084, 42.0%	< 0.001
Tobacco history	2727, 18.2%	1227, 16.0%	1500, 20.4%	< 0.001
Hypertension	6613, 44.0%	3395, 44.3%	3218, 43.8%	0.56
Dyslipidemia	6490, 43.2%	3523, 45.9%	2967, 40.4%	< 0.001
Diabetes	2417, 16.1%	1240, 16.2%	1177, 16.0%	0.75
Coronary artery disease	3082, 20.5%	1578, 20.6%	1504, 20.5%	0.87
Heart failure	1484, 9.9%	716, 9.3%	768, 10.4%	0.02
Cerebrovascular disease	1149, 7.7%	643, 8.4%	506, 6.9%	< 0.001
Chronic kidney disease	1182, 7.9%	601, 7.8%	581, 7.9%	0.87
End stage renal disease	202, 1.3%	112, 1.5%	90, 1.2%	0.21

Abbreviations: PRE, Pre‐Implementation; POST, Post‐Implementation.

The POST group had fewer troponin tests completed compared to the PRE group (mean 2.2 vs. 2.4, *p* < 0.001). Troponin elevations were detected more frequently in the POST group (24.1% vs. 13.6%), a difference which persisted in an adjusted logistic regression model (aOR = 2.71, 95% CI: 2.45, 3.00). About one‐third of patients in the POST group (35.5%) had all troponin values below the LOD ( < 6 ng/L). The final diagnostic classifications across PRE/POST groups are summarized in Table 2. An ultimate diagnosis of acute MI was documented in 906 (11.8%) patients in the PRE and 906 (12.3%) patients in the POST group. Among the 1812 MI diagnoses, 596 (32.9%) were Type 1 STEMI, 1163 (64.2%) were Type 1 NSTEMI, and 53 (2.9%) were Type 2 MI. In adjusted models, Type 1 NSTEMIs were slightly more frequent in the POST versus PRE group (aOR = 1.17; 95% CI: 1.02, 1.33, *p* = 0.02), but no differences were observed for Type 1 STEMI (aOR = 1.00; 95% CI: 0.83, 1.19, *p* = 0.95), or Type 2 MI (aOR = 1.00; 95% CI: 0.58, 1.74, *p* = 0.98). Troponin elevations without MI were observed in 1003 (7.6%) patients and were more frequent in the POST than PRE group (11.7% vs. 1.8%; aOR = 9.03; 7.44, 10.96).

**Table 2:** Diagnostic classification of patients presenting to the emergency department with a primary complaint of chest pain stratified by pre‐ versus post‐implementation of a high‐sensitivity troponin protocol.

	All (*n* = 15 015)	PRE (*n* = 7668)	POST (*n* = 7347)	*p*‐value
Troponin > 99th percentile	2815, 18.7%	1047, 13.6%	1768, 24.1%	< 0.001
OR (95% CI)^a^		1.00 (‐)	2.71 (2.45, 3.00)	< 0.001
Type 1 MI	1759, 11.7%	879, 11.5%	880, 12.0%	0.33
OR (95% CI)^a^		1.00 (‐)	1.08 (0.96, 1.20)	0.20
Type 1 STEMI	596, 4.0%	319, 4.2%	277, 3.8%	0.22
OR (95% CI)^a^		1.00 (‐)	1.00 (0.83, 1.19)	0.95
Type 1 NSTEMI	1163, 7.7%	560, 7.3%	603, 8.2%	0.04
OR (95% CI)^a^		1.00 (‐)	1.17 (1.02, 1.33)	0.02
Type 2 MI	53, 0.4%	27, 0.4%	26, 0.4%	0.99
OR (95% CI)^a^		1.00 (‐)	1.00 (0.58, 1.74)	0.98
Myocardial Injury without MI	1003, 6.7%	141, 1.8%	862, 11.7%	< 0.001
OR (95% CI)^a^		1.00 (‐)	9.03 (7.44, 10.96)	< 0.001

Abbreviations: OR, Odds Ratio; MI, Myocardial Infarction; PRE, Pre‐Implementation; POST, Post‐Implementation.

^a^ Adjusted for age, sex, race, obesity, tobacco history, hypertension, dyslipidemia, diabetes, coronary artery disease, heart failure, cerebrovascular disease, chronic kidney disease, and end‐stage renal disease.

### 3.1 Cardiac Testing at the Index Emergency Department Encounter

In adjusted models, patients in the POST group were less likely to have any index ED encounter‐related cardiac diagnostic testing (aOR = 0.72; 95% CI: 0.66, 0.77) compared to the PRE group. Specifically, the likelihood of any noninvasive testing (aOR = 0.72, 95% CI: 0.67, 0.78), including echocardiography (aOR = 0.85, 95% CI: 0.78, 0.92), stress testing (aOR = 0.76, 95% CI: 0.69, 0.82), or CCTA (aOR = 0.69, 95% CI: 0.54, 0.87) was lower (Table 3). There was no difference across groups in the performance of invasive coronary angiography (OR = 1.09 [POST vs. PRE], 95% CI: 0.97, 1.22). Diagnostic testing occurred more often in patients with elevated troponin in both PRE and POST groups (PRE: OR = 9.33, 95% CI: 7.81–11.14, *p* < 0.001: POST: OR = 10.63, 95% CI: 9.36–12.08, *p* < 0.001) (Supporting Information S1: Table 1). While echocardiograms and invasive angiography were more likely done in those with elevated troponin in both PRE and POST groups, stress testing and CCTA were less likely in the presence of a troponin elevation in the PRE group (OR = 0.23 and 0.28, respectively). In contrast, in the POST phase, stress tests were more frequent (OR = 1.54, 95% CI: 1.35, 1.75) and CCTA was performed with similar frequency (OR = 0.99, 95% CI: 0.65, 1.52) in those with elevated Hs‐trop relative to normal troponin. No significant differences were noted in index admission testing between the quaternary hospital (AGH) and community hospitals within AHN (data not shown).

**Table 3:** Performance of cardiac diagnostic testing among patients presenting to the emergency department with a primary complaint of chest pain stratified by pre‐ versus post‐implementation of a high‐sensitivity troponin protocol.

	All (*n* = 15 015)	PRE (*n* = 7668)	POST (*n* = 7347)	*p*‐value
Any cardiac diagnostic testing^a^	6201, 41.3%	3418, 44.6%	2783, 37.9%	< 0.001
OR (95% CI)^b^		1.00 (‐)	0.72 (0.66, 0.77)	< 0.001
Any noninvasive testing^c^	6047, 40.3%	3334, 43.5%	2713, 36.9%	< 0.001
OR (95% CI)^b^		1.00 (‐)	0.72 (0.67, 0.78)	< 0.001
Coronary angiography	1682, 11.2%	841, 11.0%	841, 11.4%	0.35
OR (95% CI)^b^		1.00 (‐)	1.09 (0.97, 1.22)	0.14
Echocardiography	4511, 30.0%	2434, 31.7%	2077, 28.3%	< 0.001
OR (95% CI)^b^		1.00 (‐)	0.85 (0.78, 0.92)	< 0.001
Stress test	3041, 20.2%	1718, 22.4%	1323, 18.0%	< 0.001
OR (95% CI)^b^		1.00 (‐)	0.76 (0.69, 0.82)	< 0.001
CCTA	305, 2.0%	184, 2.4%	121, 1.6%	0.001
OR (95% CI)^b^		1.00 (‐)	0.69 (0.54, 0.87)	< 0.001

Abbreviations: CCTA, Coronary Computed Tomography Angiography; OR, Odds Ratio; PRE, Pre‐Implementation; POST, Post‐Implementation.

^a^ Any of invasive coronary angiography, echocardiography, stress test, or CCTA.

^b^ Adjusted for age, sex, race, obesity, tobacco history, hypertension, dyslipidemia, diabetes, coronary artery disease, heart failure, cerebrovascular disease, chronic kidney disease, and end‐stage renal disease.

^c^ Any of echocardiography, stress test, or CCTA.

### 3.2 Emergency Department Disposition

POST patients were more likely to be directly discharged from the ED (n = 4453, 60.7% vs. *n* = 3996, 52.1%, *p* < 0.001), a difference which persisted after adjustment (aOR=1.60, 95% CI: 1.48, 1.74) (Figure 1). Median time spent in the ED was slightly longer POST (median [IQR] hours: 3.8 [2.5, 5.5] vs. 3.6 [2.4, 5.0]; *p* < 0.001). POST patients were less likely to be triaged to an observation unit (30.2% vs. 40.0%, *p* < 0.001), but no difference was observed across groups in the proportion of patients ultimately admitted to the hospital (PRE: 10.7% vs. POST: 11.4%, *p* = 0.13). Furthermore, among the 1660 (11.1%) patients admitted to the hospital, LOS in the hospital was not significantly different across groups (median [IQR] hours: PRE: 60.6 [36.4, 120.3]; POST: 55.3 [33.0, 113.9], *p* = 0.83).

### 3.3 Post‐Discharge Outcomes in Early Discharge

Among the 12 200 patients with neither MI nor troponin elevation at the index ED visit, 8045 were discharged directly from the ED (PRE *n* = 3890; POST *n* = 4155). Among these patients, five new MIs (0.06%) were diagnosed within 30 days of discharge [PRE: 4, 0.10%; POST: 1, 0.02%; *p* = 0.16], with no additional MIs up to 90 days following discharge (Figure 2). Furthermore, within 30 days of discharge 2 deaths from any cause occurred (one each in the PRE and POST periods). Within 90 days of discharge, there were 22 total deaths (12 in PRE, 10 in POST, *p* = 0.56). There was a significant decrease in 30‐day return ED visits after discharge in the POST group compared to the PRE group (6.4% vs. 8.9%, aOR = 0.70 (0.59, 0.83), *p* < 0.001). This difference persisted in the 90‐day period (aOR = 0.70 (0.62, 0.80), *p* < 0.001).

### 3.4 Post‐Discharge Healthcare Utilization and Costs

Among the 15 015 patients from the original study cohort, 4516 (30%) were continuously enrolled in the HHP both 3 months before the index ED presentation and 3 months following discharge. The baseline characteristics of this subset were largely similar to the original cohort (Supporting Information S1: Table 2). Overall costs were slightly though not significantly lower among POST compared to PRE patients (median [IQR] costs per member per month: 824 [258, 2524] vs. 868 [291, 2458], *p* = 0.30) (Supporting Information S1: Table 3). In an adjusted model for overall costs, POST costs were not different from PRE costs (‐3.2% lower in POST vs. PRE; 95% CI: ‐5.9%, 12.2%, *p* = 0.49). When separated by different claim types, costs for professional services (‐8.8%; 95% CI: ‐16.9%, ‐1.0%, *p* = 0.03) and drug prescriptions (‐15.2%; 95% CI: ‐24.0%, ‐6.4%, *p* < 0.001) were significantly lower in the POST group, while no significant cost differences were observed related to outpatient (‐7.9%; 95% CI: ‐24.7%, 8.9%, *p* = 0.36) or inpatient ( + 10.7%; 95% CI: ‐5.2%, 26.6%, *p* = 0.19) claims. Overall healthcare utilization (i.e., number of encounters) was significantly lower in the POST compared to PRE group (median [IQR]: 5 [3,11] vs. 6 [3, 11], *p* < 0.001) (Supporting Information S1: Table 4). In an adjusted model, POST encounters were ‐8.4% (95% CI: ‐4.1%, ‐12.8%, *p* < 0.001) less frequent compared to PRE (*p* < 0.001). When separated by claim type, professional services (‐7.8%; 95% CI: ‐3.0%, ‐12.6%, *p* = 0.002) and drug prescriptions (‐3.5%; 95% CI: ‐7.1%, 0.2%, *p* = 0.06) were (nearly) significantly less frequent in the POST group, while outpatient ( + 0.9%, 95% CI: ‐2.8%, 4.6%, *p* = 0.63) and inpatient encounters (‐1.2%, 95% CI: ‐2.7%, 0.4%, *p* = 0.13) were not different.

## 4 Discussion

We report on the transition to the high‐sensitivity Roche Gen‐5 troponin‐T assay within an integrated health network in western Pennsylvania with specific focus on index hospital resource utilization in addition to post‐discharge outcomes, resource utilization, and costs. In a subset of study patients with accessible insurance claims data, we demonstrate that this transition led to decreased overall healthcare utilization and lower costs of care with respect to professional claims and drug prescriptions. This was accompanied by lower rates of return ED visits at 30 and 90 days, and low rates of MI in those discharged directly from the ED. This is the first report of extended follow up data and cost of care after transition to a high‐sensitivity troponin T‐based chest pain pathway in a large US healthcare system consisting of academic and community hospitals.

We attribute the successful implementation of our high‐sensitivity troponin protocol to many factors: multi‐stakeholder engagement and active involvement in institutional protocol development with extensive education, regular feedback to providers, availability of a clinical decision support tool to encourage and facilitate proper application of the protocol, and an incentivized organization focused on controlling costs. This is in line with recommendations from the 2021 ACC Chest Pain guidelines advocating transition to Hs‐trop [3]. Our study was able to replicate previously reported findings of more frequent ED discharges and greater diagnosis of myocardial injury with a high‐sensitivity troponin protocol [14]. This must be balanced with more frequent troponin elevations with the Hs‐troponin T assay which may reflect chronic troponin elevations that occur in settings such as heart failure, arrhythmias, and certain noncardiac conditions such as renal failure, anemia, and sepsis. The concern has been raised that the higher sensitivity assay may lead to additional testing due to higher frequency of abnormal test results. We demonstrate that in a system that includes a large academic medical center as well as several community hospitals, the rate of noninvasive ischemic testing such as stress testing and CCTA actually declined in the POST phase. Moreover, testing occurred infrequently in those with troponin levels < 99th percentile, likely reflecting confidence in negative test results. With the Gen‐4 troponin assay, stress testing and CCTA were performed more frequently among those with normal than elevated troponin findings. In contrast, with the Hs‐trop assay, stress testing was performed more frequently among those with elevated troponin, specifically in patients with myocardial injury, reflecting the clinician uncertainty in test interpretation among patients with indeterminate biomarker results. As emphasized in prior studies, transition to the high‐sensitivity assay requires multi‐stakeholder engagement, creation of a protocol, and extensive education of providers, nurses, trainees, and laboratory personnel. Education is an integral part of this process to ensure confidence among providers that a negative result implies extremely low risk in a low pretest probability patient and hence discharge from the ED is appropriate. Moreover, further cardiac testing in this low‐risk population may not be warranted as avoidance of testing as demonstrated in our study did not lead to worse outcomes in the 90‐day follow up period.

Our additional analysis of 30‐day outcomes including death, new MI, ED return visit, and hospitalization is reassuring that the higher percentage of discharges in the POST period did not result in higher rates of adverse outcomes. There were no differences in the number of MIs or deaths in 30‐ and 90‐day follow up among early discharge patients across PRE and POST phases and rates were consistently < 1%. This is the allowable threshold for missed MI proposed by the American College of Emergency Physicians [15]. This should give additional confidence to ED physicians and cardiologists considering transition to the Hs‐troponin T assay. Despite the known low adverse cardiac event rate among patients who rule out for MI even with previous troponin assays, there is reluctance to discharge these patients from the ED. The national admission rate for chest pain patients is 14.0% (5.5‐27.8%) and there is great variability between institutions that depends largely on local practice patterns [16, 17]. Moreover, higher rates of admission do not correlate with better rates of 30‐day MI or death. Prior studies have shown that the US healthcare system can save up to $3 billion annually by reducing variation in ED hospitalization for chest pain [18]. Concerns about litigation and lack of longer follow up data in patients discharged from the ED are some reasons preventing practice change in the US [19].

Health systems also have concerns about increased utilization that could potentially result from implementation of a higher sensitivity assay that yields greater numbers of abnormal results [20]. Mean costs of care for chest pain evaluation in the ED in 2016 was estimated at US$6325, with a total annual cost of US$1.5 billion [21]. As a combined payer‐provider, our health network is uniquely positioned to obtain information on costs due to availability of claims data. We clearly demonstrate lower utilization not only at the index ED encounter but also in the first 3 months of follow up in the POST group. Although a concomitant reduction in overall costs was not evident in an adjusted model, costs related to professional charges and drug prescriptions were significantly lower in the POST period. This likely reflects the lower rates of testing in the follow up period although our claims data could not separate costs specifically related to individual testing. Lack of differences in inpatient and outpatient costs despite lower rates of utilization in the POST group may relate to differences in financial contracts between the two time periods. With health systems transitioning to value‐based reimbursement models such as global capitation, the above findings reflecting decreased cost of care should be reassuring. Since the study was restricted to patients with a diagnosis code indicative of chest pain, troponin measurements that may have occurred in non‐chest pain scenarios were excluded. Hence, the study did not assess the downstream implications of troponins measured in these settings.

The current study specifically focused on the post‐discharge healthcare costs as this is a vulnerable period that can be associated with adverse outcomes and higher utilization/costs. Chuang et al. reported on posthospital outcomes and costs in Australia and showed that a 0/1‐h Hs‐trop protocol did not lead to additional cost savings compared to a 0/3‐h Hs‐trop protocol [6]. A report from the National Health and Care Research analyzed the economic implications of transition to Hs‐trop assays by assessing the incremental cost‐effectiveness ratio (ICER) [22]. Compared with standard troponin testing, Hs‐trop testing resulted in probabilistic ICERs ranging between £34307 and £36 842 603 in savings per QALY lost. These numbers derived from economic modeling of multiple European studies, some of which included outpatient costs, may not reflect US healthcare practices. Ganguli et al. reported lower downstream utilization after Hs‐trop implementation along with less healthcare spending during the index hospitalization in those presenting with chest pain [3]. In contrast, an early report on hospital costs by Haneke et al showed higher costs of care [23]. The lower utilization during index hospitalization that seems to continue up to 90 days post‐discharge along with a trend towards lower costs is the primary finding in the current study. This coupled with the lack of increase in adverse outcomes within 30 and 90 days should provide assurance to health systems considering transition to Hs‐troponin.

### 4.1 Limitations

While this study provides a comprehensive assessment of an Hs‐troponin T implementation project, it reflects the experience of one health care system and its local practice patterns. However, the principles discussed here should be applicable to other hospitals but individual variations and institutional culture may produce dissimilar results. It is important to note that the results described here are only applicable to the Roche Gen‐5 troponin T assay and while similar, the Abbott and other assays of high‐sensitivity troponin‐I may not yield the same results. Our study design did not include all patients in whom troponin assays were completed but rather included those discharged or admitted with a chest pain diagnosis based on billing codes which may have inherent misclassification. However, we only included patients where this was a primary diagnosis to increase accuracy. This inclusion criteria may have missed other presentations which could have been angina equivalents although a previous study showed no differences in downstream testing or new diagnoses when troponin testing was done in non‐chest pain scenarios [3]. Our study was unique in categorization of elevated troponin into Type 1 and Type 2 MI and non‐MI‐related troponin elevation. Our hospital system led a large educational initiative to avoid misclassification of chronic myocardial injury as Type 2 MI as well as avoiding documentation of Type 2 MI as NSTEMI. For study purposes, categorization of Type 1 and 2 MI was based on ICD‐10 billing codes rather than chart review which may have led to misclassification. However, the same methodology was applied in both PRE and POST phases which lends confidence to our results. Although HEART scores were part of the chest pain diagnostic pathways in the PRE and POST phases, scores were not consistently documented in the EHR and hence not included in the analysis. Recent Hs‐trop‐based triage pathways in Europe have not included any risk score and the utility of scores in these new pathways is currently debated. The recent 2022 ACC/AHA ED chest pain guideline makes reference to this question but does not provide clear guidance on negating or modifying the score. Moreover, our paper was focused on the downstream clinical and economic implications of the Hs‐troponin transition since several previous publications focused on predictive metrics of an Hs‐trop protocol. While we were able to obtain cost data in the study period, we did not have information on the contracted rates for different testing, inpatient, and outpatient visits. In addition, location of testing, hospital‐based versus office‐based could also account for different costs/payments. Nevertheless, the lack of increase in costs with transition to the Hs‐trop protocol is the primary result.

In conclusion, transition to an Hs‐trop pathway for evaluation of chest pain was associated with a higher frequency of direct ED discharge and lower rates of noninvasive cardiac testing without any change in the rate of invasive coronary angiography. The 30‐ and 90‐day outcomes of patients directly discharged from the ED demonstrate adverse event rates < 1% and a decline in return ED visits post‐implementation. Healthcare utilization continued to be lower within 90‐day follow up in the post‐implementation group but this did not translate into clear reductions in healthcare costs.

## Conflicts of Interest

The authors declare no conflicts of interest.

## Supporting information

HsTrop manuscript Supplemental Tables ClinCardiol.

Supplemental Figure 1. Chest Pain Assessment Pathway Prior to the Implementation of a High‐Sensitivity Troponin Protocol. Supplemental Figure 2. AHN (Allegheny Health Network) ED High‐Sensitivity Troponin Interpretation.

## Data Availability

The data that support the findings of this study are available from Highmark Health Plan. Restrictions apply to the availability of these data, which were used under license for this study. Data are available from the author(s) with the permission of Highmark Health Plan. The data that support the findings of this study are available on request from the corresponding author. The data are not publicly available due to privacy or ethical restrictions.

## References

[1] A. R. Chapman , P. D. Adamson , A. S. V. Shah , et al., “High‐Sensitivity Cardiac Troponin and the Universal Definition of Myocardial Infarction,” Circulation 141 (2020): 161–171. 31587565 10.1161/CIRCULATIONAHA.119.042960 PMC6970546

[2] J. L. Januzzi, Jr. , S. A. Mahler , R. H. Christenson , et al., “Recommendations for Institutions Transitioning to High‐Sensitivity Troponin Testing,” Journal of the American College of Cardiology 73 (2019): 1059–1077. 30798981 10.1016/j.jacc.2018.12.046

[3] M. Gulati , P. D. Levy , D. Mukherjee , et al., “2021 AHA/ACC/ASE/CHEST/SAEM/SCCT/SCMR Guideline for the Evaluation and Diagnosis of Chest Pain: A Report of the American College of Cardiology/American Heart Association Joint Committee on Clinical Practice Guidelines,” Circulation 144 (2021): e368–e454. 10.1161/CIR.0000000000001029 34709879

[4] C. McCarthy , S. Li , T. Y. Wang , et al., “Implementation of High‐Sensitivity Cardiac Troponin Assays in the United States,” Journal of the American College of Cardiology 81 (2023): 207–219. 36328155 10.1016/j.jacc.2022.10.017 PMC10037558

[5] T. R. Johannessen , S. Halvorsen , D. Atar , et al., “Cost‐Effectiveness of a Rule‐Out Algorithm of Acute Myocardial Infarction in Low‐Risk Patients: Emergency Primary Care Versus Hospital Setting,” BMC Health Services Research 22 (2022): 1274. 36271364 10.1186/s12913-022-08697-6 PMC9587629

[6] M. A. Chuang , E. S. Gnanamanickam , J. Karnon , et al., “Cost Effectiveness of a 1‐Hour High‐Sensitivity Troponin‐T Protocol: An Analysis of the RAPID‐TnT Trial,” International Journal of Cardiology. Heart & Vasculature 38 (2022): 100933. 35024428 10.1016/j.ijcha.2021.100933 PMC8728427

[7] P. Jülicher , J. H. Greenslade , W. A. Parsonage , and L. Cullen , “The Organisational Value of Diagnostic Strategies Using High‐Sensitivity Troponin for Patients With Possible Acute Coronary Syndromes: A Trial‐Based Cost‐Effectiveness Analysis,” BMJ Open 7 (2017): e013653. 10.1136/bmjopen-2016-013653 PMC5577894 28601817

[8] I. Ganguli , J. Cui , N. Thakore , et al., “Downstream Cascades of Care Following High‐Sensitivity Troponin Test Implementation,” Journal of the American College of Cardiology 77 (2021): 3171–3179. 34167642 10.1016/j.jacc.2021.04.049 PMC13572992

[9] R. Vigen , D. B. Diercks , I. A. Hashim , et al., “Association of a Novel Protocol for Rapid Exclusion of Myocardial Infarction With Resource Use in a US Safety Net Hospital,” JAMA Network Open 3 (2020): e203359. 32320036 10.1001/jamanetworkopen.2020.3359 PMC7177202

[10] T. J. Judson , L. Y. Beach , and K. Soni , “The Troponin Cascade: A Teachable Moment,” JAMA Internal Medicine 177 (2017): 1193–1194. 28558102 10.1001/jamainternmed.2017.1804

[11] J. W. Mold and H. F. Stein , “The Cascade Effect in the Clinical Care of Patients,” New England Journal of Medicine 314 (1986): 512–514. 3945278 10.1056/NEJM198602203140809

[12] https://www.cms.gov/priorities/innovation/key-concepts/risk-based-arrangements-health-care.

[13] K. Thygesen , J. S. Alpert , A. S. Jaffe , et al., “Fourth Universal Definition of Myocardial Infarction (2018),” Journal of the American College of Cardiology 72 (2018): 2231–2264. 30153967 10.1016/j.jacc.2018.08.1038

[14] O. Ola , A. Akula , L. De Michieli , et al., “Clinical Impact of High‐Sensitivity Cardiac Troponin T Implementation in the Community,” Journal of the American College of Cardiology 77 (2021): 3160–3170. 34167641 10.1016/S0735-1097(21)04515-0 PMC9090513

[15] M. Than , M. Herbert , D. Flaws , et al., “What Is an Acceptable Risk of Major Adverse Cardiac Event in Chest Pain Patients Soon After Discharge From the Emergency Department?,” International Journal of Cardiology 166 (2013): 752–754. 23084108 10.1016/j.ijcard.2012.09.171

[16] S. Natsui , B. C. Sun , E. Shen , et al., “Higher Emergency Physician Chest Pain Hospitalization Rates Do Not Lead to Improved Patient Outcomes,” Circulation: Cardiovascular Quality and Outcomes 14 (2021): e006297. 33430609 10.1161/CIRCOUTCOMES.119.006297 PMC7855368

[17] K. Caines , C. Shoff , D. M. Bott , and J. M. Pines , “County‐Level Variation in Emergency Department Admission Rates Among US Medicare Beneficiaries,” Annals of Emergency Medicine 68 (2016): 456–460. 27085370 10.1016/j.annemergmed.2016.03.019

[18] A. K. Sabbatini , B. K. Nallamothu , and K. E. Kocher , “Reducing Variation in Hospital Admissions From the Emergency Department for Low‐Mortality Conditions May Produce Savings,” Health Affairs 33 (2014): 1655–1663. 25201672 10.1377/hlthaff.2013.1318

[19] J. M. Pines , J. A. Isserman , D. Szyld , et al., “The Effect of Physician Risk Tolerance and the Presence of an Observation Unit on Decision Making for ED Patients With Chest Pain,” American Journal of Emergency Medicine 28 (2010): 771–799. 20837253 10.1016/j.ajem.2009.03.019

[20] A. T. Sandhu , P. A. Heidenreich , J. Bhattacharya , and M. K. Bundorf , “Cardiovascular Testing and Clinical Outcomes in Emergency Department Patients With Chest Pain,” JAMA Internal Medicine 177 (2017): 1175–1182. 28654959 10.1001/jamainternmed.2017.2432 PMC5710427

[21] A. A. Aalam , A. Alsabban , and J. M. Pines , “National Trends in Chest Pain Visits in US Emergency Departments (2006–2016),” Emergency Medicine Journal 37 (2020): 696–699. 32900857 10.1136/emermed-2020-210306

[22] M. Westwood , B. Ramaekers , S. Grimm , et al., “High‐Sensitivity Troponin Assays for Early Rule‐Out of Acute Myocardial Infarction in People With Acute Chest Pain: A Systematic Review and Economic Evaluation,” Health Technology Assessment 25 (2021): 1–276. 10.3310/hta25330 PMC8200931 34061019

[23] T. W. Haneke , R. J. Widmer , E. M. Fry , G. M. Wilson , and J. B. Michel , “Hospital Charges Associated With Inpatient Troponin Testing,” Journal of the American College of Cardiology 72, no. 23 Pt A (2018): 2940. 30522659 10.1016/j.jacc.2018.09.053

