# Supplementary material for: Clinical Outcomes, Healthcare Utilization, and Cost Following Implementation of a High‐Sensitivity Cardiac Troponin Assay
Source: Clin Cardiol. 2025 May 6;48(5):e70133. doi: 10.1002/clc.70133 (PMC12053897; doi:10.1002/clc.70133)
Supplement: Supplementary file 1 — HsTrop manuscript Supplemental Tables ClinCardiol. [file CLC-48-e70133-s002.docx]

Supplemental Table 1. Cardiac Testing and Emergency Department Disposition by Elevated vs. Normal Troponin Results Pre- and Post-Implementation of a High-Sensitivity Troponin Protocol

|  | PRE | | POST | |
| --- | --- | --- | --- | --- |
|  | Normal Troponin^1^  (n=6621) | Elevated Troponin^1^  (n=1047) | Normal Troponin^2^  (n=5579) | Elevated Troponin^2^  (n=1768) |
| Any cardiac diagnostic testing^3^  OR (95% CI) | 2526, 38.2%  1.00 (-) | 892, 85.2%  9.33 (7.81, 11.14) | 1402, 25.1%  1.00 (-) | 1381, 78.1%  10.63 (9.36, 12.08) |
| Any noninvasive testing^4^  OR (95% CI) | 2505, 37.8%  1.00 (-) | 829, 79.2%  6.25 (5.34, 7.31) | 1385, 24.8%  1.00 (-) | 1328, 75.1%  9.14 (8.08, 10.34) |
| Coronary angiography  OR (95% CI) | 109, 1.6%  1.00 (-) | 732, 69.9%  138.83 (110.22, 174.88) | 77, 1.4%  1.00 (-) | 764, 43.2%  54.37 (42.61, 69.39) |
| Echocardiography  OR (95% CI) | 1627, 24.6%  1.00 (-) | 807, 77.1%  10.32 (8.84, 12.05) | 924, 16.6%  1.00 (-) | 1153, 65.2%  9.44 (8.37, 10.66) |
| Stress test  OR (95% CI) | 1645, 24.8%  1.00 (-) | 73, 7.0%  0.23 (0.18, 0.29) | 914, 16.4%  1.00 (-) | 409, 23.1%  1.54 (1.35, 1.75) |
| CCTA  OR (95% CI) | 176, 2.7%  1.00 (-) | 8, 0.8%  0.28 (0.14, 0.57) | 92, 1.6%  1.00 (-) | 29, 1.6%  0.99 (0.65, 1.52) |
| Direct ED discharge  OR (95% CI) | 3890, 58.8%  1.00 (-) | 106, 10.1%  0.08 (0.06, 0.10) | 4155, 74.5%  1.00 (-) | 298, 16.9%  0.07 (0.06, 0.08) |

^1^ Elevated troponin for Gen-4 assay defined as values ≥0.01 ng/ml for both sexes

^2^ Elevated troponin for Gen-5 assay defined as exceeding the sex-specific 99th percentile of the assay (14 ng/L for women; 22 ng/L for men)

^3^ Any of invasive coronary angiography, echocardiography, stress test, or CCTA

^4^ Any of echocardiography, stress test, or CCTA

CCTA = Coronary Computed Tomography Angiography; OR = Odds Ratio

PRE=Pre-Implementation; POST=Post-Implementation

Supplemental Table 2: Baseline Characteristics of Patients Presenting to the Emergency Department with a Primary Complaint of Chest Pain Stratified by Pre- vs. Post-Implementation of a High-Sensitivity Troponin Protocol, Restricted to Patients Enrolled in the Highmark Health Plan

|  | All | PRE | POST |  |
| --- | --- | --- | --- | --- |
|  | (n=4516) | (n=2262) | (n=2254) | p-value |
| Age | 57.8 (45.0, 70.3) | 58.6 (45.6, 70.6) | 57.1 (44.1, 69.7) | 0.07 |
| Female | 2,539 (56%) | 1,285 (57%) | 1,254 (56%) | 0.44 |
| White race | 3,875 (86%) | 1,926 (85%) | 1,949 (86%) | 0.22 |
| Obesity | 1,889 (42%) | 907 (40%) | 982 (44%) | 0.02 |
| Tobacco history | 576 (13%) | 252 (11%) | 324 (14%) | 0.001 |
| Hypertension | 2,274 (50%) | 1,137 (50%) | 1,137 (50%) | 0.93 |
| Dyslipidemia | 2,251 (50%) | 1,206 (53%) | 1,045 (46%) | <0.001 |
| Diabetes | 774 (17%) | 389 (17%) | 385 (17%) | 0.95 |
| Coronary artery disease | 1,094 (24%) | 549 (24%) | 545 (24%) | 0.97 |
| Heart failure | 466 (10%) | 211 (9.3%) | 255 (11%) | 0.03 |
| Cerebrovascular disease | 364 (8.1%) | 202 (8.9%) | 162 (7.2%) | 0.04 |
| Chronic kidney disease | 387 (8.6%) | 196 (8.7%) | 191 (8.5%) | 0.86 |
| End stage renal disease | 44 (1.0%) | 26 (1.1%) | 18 (0.8%) | 0.29 |

PRE=Pre-Implementation; POST=Post-Implementation

Supplemental Table 3. Healthcare Costs During 3 Months Following Emergency Department Discharge Pre- vs. Post-Implementation of a High-Sensitivity Troponin Protocol, Restricted to Patients Enrolled in the Highmark Health Plan

|  | All  (n=4516) | PRE  (n=2262) | POST  (n=2254) | p-value |
| --- | --- | --- | --- | --- |
| Overall Costs  Median (IQR)  Mean (SD)  Unadjusted β (95% CI)  Adjusted β^1^ (95% CI) | 851 (273, 2482)  5406 (22227)  -  - | 868 (291, 2458)  5132 (20710)  Ref  Ref | 824 (258, 2524)  5682 (23653)  -6.5% (-17.1, 4.2)  -3.2% (-12.2, 5.9) | 0.30  0.23  0.49 |
| Outpatient  Median (IQR)  Mean (SD)  Unadjusted β (95% CI)  Adjusted β^1^ (95% CI) | 109 (0, 779)  775 (1580)  -  - | 110 (0, 757)  754 (1577)  Ref  Ref | 108 (0, 800)  776 (1583)  -11.5% (-29.3, 6.3)  -7.9% (-24.7, 8.9) | 0.20  0.21  0.36 |
| Professional  Median (IQR)  Mean (SD)  Unadjusted β (95% CI)  Adjusted β^1^ (95% CI) | 345 (146, 778)  675 (891)  -  - | 353 (158, 787)  686 (886)  Ref  Ref | 336 (133, 766)  668 (896)  -10.8% (-20.0 -1.7)  -8.8% (-16.9, -1.0) | 0.07  0.02  0.03 |
| Inpatient  Median (IQR)  Mean (SD)  Unadjusted β (95% CI)  Adjusted β^1^ (95% CI) | 0 (0, 0)  3383 (21213)  -  - | 0 (0, 0)  3116 (19674)  Ref  Ref | 0 (0, 0)  3651 (22654)  +4.8% (-12.0, 21.6)  +10.7% (-5.2, 26.6) | 0.60  0.58  0.19 |
| Drug Prescriptions  Median (IQR)  Mean (SD)  Unadjusted β (95% CI)  Adjusted β^1^ (95% CI) | 36 (2, 291)  286 (601)  -  - | 41 (3, 330)  283 (582)  Ref  Ref | 30 (0, 267)  288 (618)  -22.3% (-37.1, -7.6)  -15.2% (-24.0, -6.4) | 0.003  0.003  <0.001 |

^1^ Adjusted for baseline costs, age, sex, race, obesity, tobacco history, hypertension, dyslipidemia, diabetes, coronary artery disease, heart failure, cerebrovascular disease, chronic kidney disease, and end-stage renal disease.

All values reported as per member per month.

Supplemental Table 4. Healthcare Utilization During 3 Months Following Emergency Department Discharge, Pre- vs. Post-Implementation of a High-Sensitivity Troponin Protocol, Restricted to Patients Enrolled in the Highmark Health Plan

|  | All  (n=4516) | PRE  (n=2262) | POST  (n=2254) | p-value |
| --- | --- | --- | --- | --- |
| Overall Utilization  Median (IQR)  Mean (SD)  Unadjusted β (95% CI)  Adjusted β^1^ (95% CI) | 6 (3, 10)  7.83 (7.01)  -  - | 6 (3, 11)  8.10 (7.06)  Ref  Ref | 5 (3, 10)  7.56 (6.96)  -11.4% (-17.3, -5.6)  -8.4% (-4.1, 12.8) | <0.001  <0.001  <0.001 |
| Outpatient  Median (IQR)  Mean (SD)  Unadjusted β (95% CI)  Adjusted β^1^ (95% CI) | 0.33 (0, 1)  0.79 (0.94)  -  - | 0.33 (0, 1)  0.82 (0.95)  Ref  Ref | 0.33 (0, 1)  0.77 (0.92)  +0.6% (-3.2, 4.5)  +0.9% (-2.8, 4.6) | 0.014  0.75  0.63 |
| Professional  Median (IQR)  Mean (SD)  Unadjusted β (95% CI)  Adjusted β^1^ (95% CI) | 2.7 (1.3, 5.3)  4.2 (4.5)  -  - | 3 (1.3, 5.7)  4.37 (4.57)  Ref  Ref | 2.7 (1.3, 5.0)  4.05 (4.43)  -9.9% (-15.7, -4.1)  -7.8% (-3.0, -12.6) | <0.001  <0.001  0.002 |
| Inpatient  Median (IQR)  Mean (SD)  Unadjusted β (95% CI)  Adjusted β^1^ (95% CI) | 0 (0, 0)  0.06 (0.28)  -  - | 0 (0, 0)  0.07 (0.29)  Ref  Ref | 0 (0, 0)  0.06 (0.26)  -0.8% (-2.4, 0.8)  -1.2% (-2.7, 0.4) | 0.80  0.35  0.13 |
| Drug Prescriptions  Median (IQR)  Mean (SD)  Unadjusted β (95% CI)  Adjusted β^1^ (95% CI) | 2.0 (0.33, 4.0)  2.66 (2.72)  -  - | 2.0 (0.33, 4.0)  2.75 (2.70)  Ref  Ref | 1.67 (0, 4)  2.58 (2.73)  -5.8% (-10.8, -0.7)  -3.5% (-7.1, 0.2) | 0.001  0.03  0.06 |

^1^ Adjusted for baseline utilization, age, sex, race, obesity, tobacco history, hypertension, dyslipidemia, diabetes, coronary artery disease, heart failure, cerebrovascular disease, chronic kidney disease, and end-stage renal disease.

All values reported as per member per month.
