# Supplementary material for: Clinical Outcomes, Healthcare Utilization, and Cost Following Implementation of a High‐Sensitivity Cardiac Troponin Assay
Source: Clin Cardiol. 2025 May 6;48(5):e70133. doi: 10.1002/clc.70133 (PMC12053897; doi:10.1002/clc.70133)
Supplement: Supplementary file 2 — Supplemental Figure 1. Chest Pain Assessment Pathway Prior to the Implementation of a High‐Sensitivity Troponin Protocol. Supplemental Figure 2. AHN (Allegheny Health Network) ED High‐Sensitivity Troponin Interpretation. [file CLC-48-e70133-s001.pptx]

## Slide 1
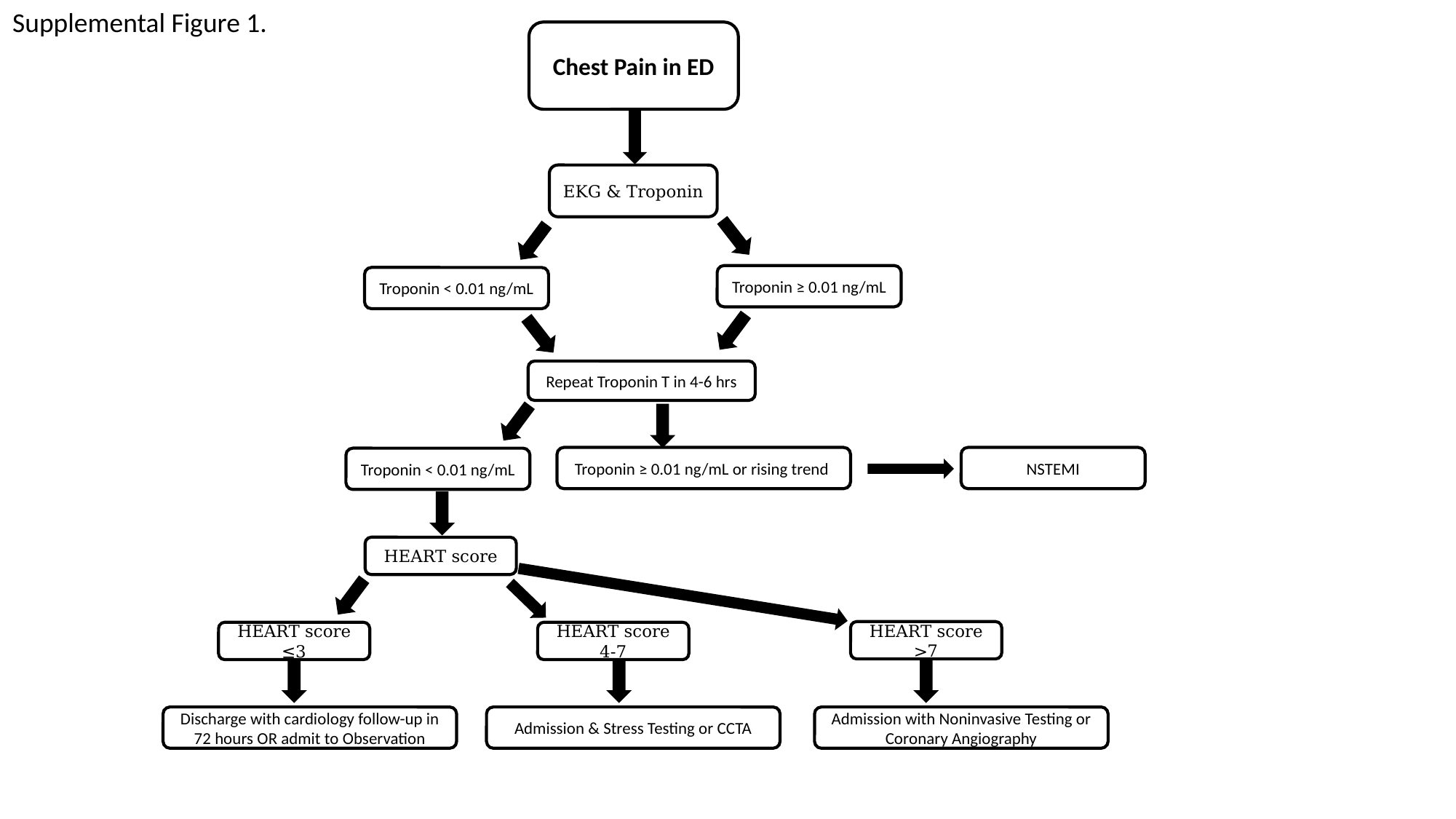

Supplemental Figure 1.
Chest Pain in ED
EKG & Troponin
Troponin ≥ 0.01 ng/mL
Troponin < 0.01 ng/mL
Repeat Troponin T in 4-6 hrs
Troponin ≥ 0.01 ng/mL or rising trend
NSTEMI
Troponin < 0.01 ng/mL
HEART score
HEART score >7
HEART score ≤3
HEART score 4-7
Discharge with cardiology follow-up in 72 hours OR admit to Observation
Admission & Stress Testing or CCTA
Admission with Noninvasive Testing or Coronary Angiography

## Slide 2
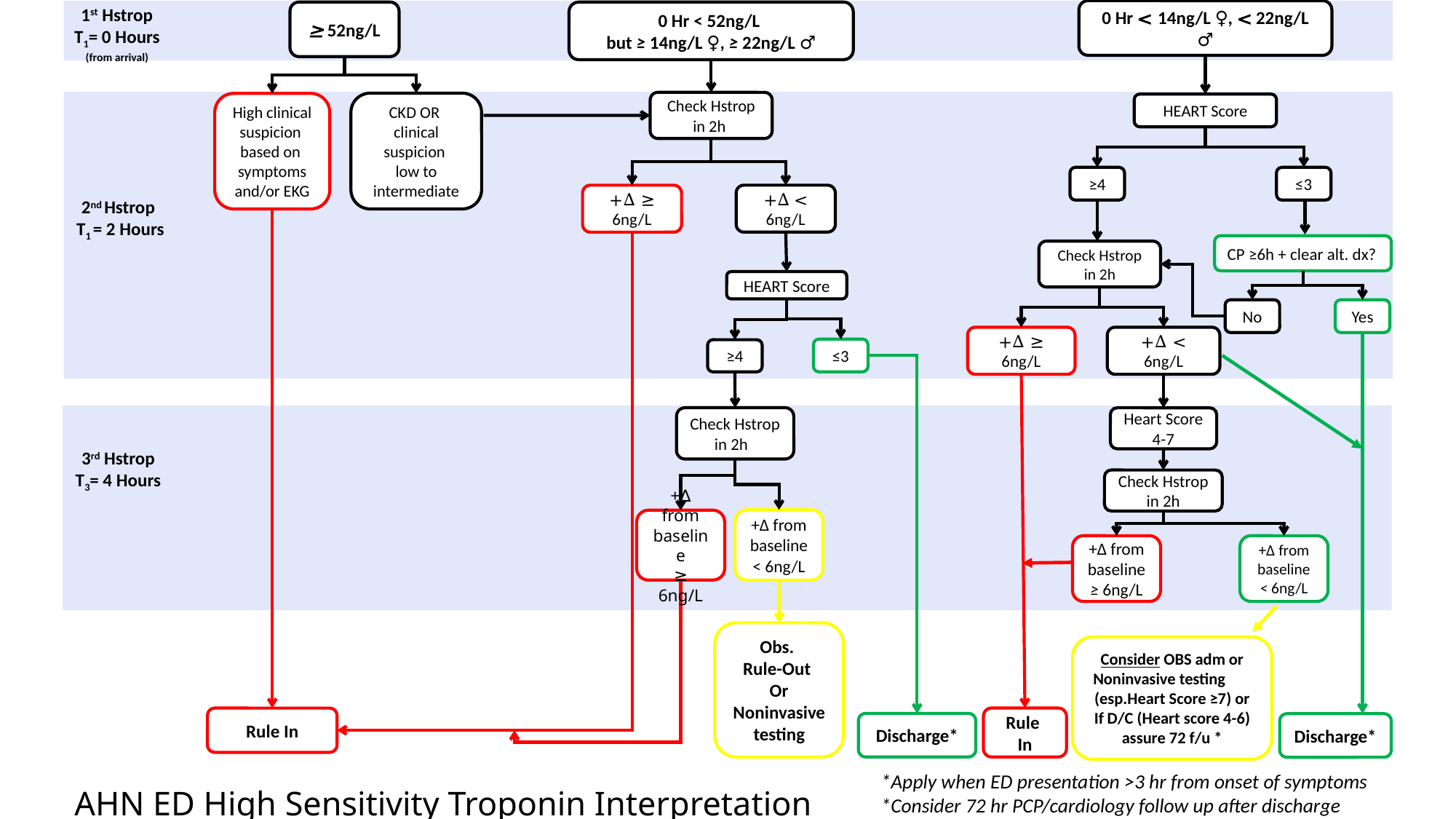

1st Hstrop
T1= 0 Hours
(from arrival)
0 Hr < 14ng/L ♀, < 22ng/L ♂
≥ 52ng/L
0 Hr < 52ng/L
but ≥ 14ng/L ♀, ≥ 22ng/L ♂
Check Hstrop
in 2h
CKD OR
clinical suspicion
low to intermediate
High clinical suspicion
based on
symptoms and/or EKG
HEART Score
2nd Hstrop
T1 = 2 Hours
≥4
≤3
+∆ ≥ 6ng/L
+∆ < 6ng/L
CP ≥6h + clear alt. dx?
Check Hstrop
in 2h
HEART Score
No
Yes
+∆ ≥ 6ng/L
+∆ < 6ng/L
≤3
≥4
3rd Hstrop
T3= 4 Hours
Check Hstrop
in 2h
Heart Score
4-7
Check Hstrop
in 2h
+∆ from baseline
< 6ng/L
+∆ from baseline
≥ 6ng/L
+∆ from baseline
≥ 6ng/L
+∆ from baseline
< 6ng/L
Obs.
Rule-Out
Or
Noninvasive testing
Consider OBS adm or Noninvasive testing (esp.Heart Score ≥7) or
If D/C (Heart score 4-6) assure 72 f/u *
Rule In
Rule
In
Discharge*
Discharge*
*Apply when ED presentation >3 hr from onset of symptoms
*Consider 72 hr PCP/cardiology follow up after discharge
AHN ED High Sensitivity Troponin Interpretation
